# Covert Optical Fingerprints Within Overt Structural Colors via Cascaded Cavity Resonances

**DOI:** 10.1002/advs.77859

**Published:** 2026-09-27

**Authors:** Gyurin Kim, Jiyeong Ma, Juhwan Kim, JuHyeong Lee, Heungchan Kwon, Young Min Song, Hyeon‐Ho Jeong

**Affiliations:** ^1^ Department of Electrical Engineering and Computer Science Gwangju Institute of Science and Technology Gwangju Republic of Korea; ^2^ School of Electrical Engineering Korea Advanced Institute of Science and Technology Daejeon Republic of Korea; ^3^ GIST InnoCORE AI‐Nano Convergence Institute for Early Detection of Neurodegenerative Diseases Gwangju Institute of Science and Technology (GIST) Gwangju Republic of Korea; ^4^ Department of Semiconductor Engineering Gwangju Institute of Science and Technology Gwangju Republic of Korea

**Keywords:** cascaded cavity resonance, monolithic fabrication, nanophotonics, optical duality, short‐range correlated disorder

## Abstract

Structural colors and stochastic optical fingerprints are difficult to integrate within a single nanophotonic platform because deterministic optical uniformity often conflicts with disorder‐driven optical randomness. Here, we introduce cascaded cavity resonances to weakly correlate and control these two optical functionalities through vertically integrated layered and nanogap cavities. In this multiscale architecture, layered photonic cavities govern overt far‐field structural coloration, whereas localized plasmonic nanogap cavities generate covert stochastic optical scattering with minimal dependence on the overt optical appearance. The proposed architecture, consisting of a Cu mirror, HfO_2_ dielectric layers, and disordered Cu nanoparticles, is fabricated entirely through a monolithic all‐vacuum deposition process. In particular, cooled glancing‐angle deposition suppresses nanoparticle coalescence during growth, enabling dense yet short‐range‐correlated nanoparticle assemblies with short nanoscale correlation lengths (∼44 nm) and high nominal stochastic encoding density exceeding 6.94×10^7^ bit/mm^2^. Leveraging these properties, we realize wafer‐scale optical physical unclonable functions containing 22,500 independently encoded optical fingerprints together with rapid authentication and robust environmental stability. These results therefore demonstrate a scalable methodology to bridge deterministic nanophotonics and stochastic security media through precision multiscale resonance engineering.

## Graphical abstract

Cascaded photonic–plasmonic cavities combine vivid structural colors with hidden stochastic optical fingerprints in a single monolithic platform. Layered cavities control the overt appearance, while disordered nanogap resonances generate covert scattering with limited mutual influence. This multiscale design enables scalable, high‐density physical unclonable functions with robust optical authentication under mechanical, aqueous, and thermal stress.

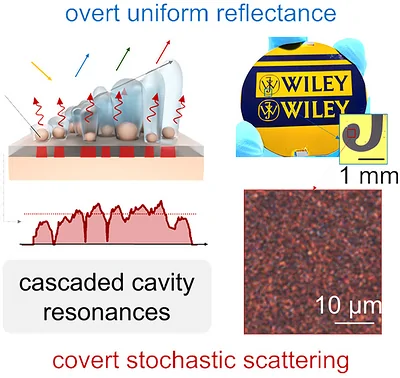

## 1 Introduction

**FIGURE 1:**
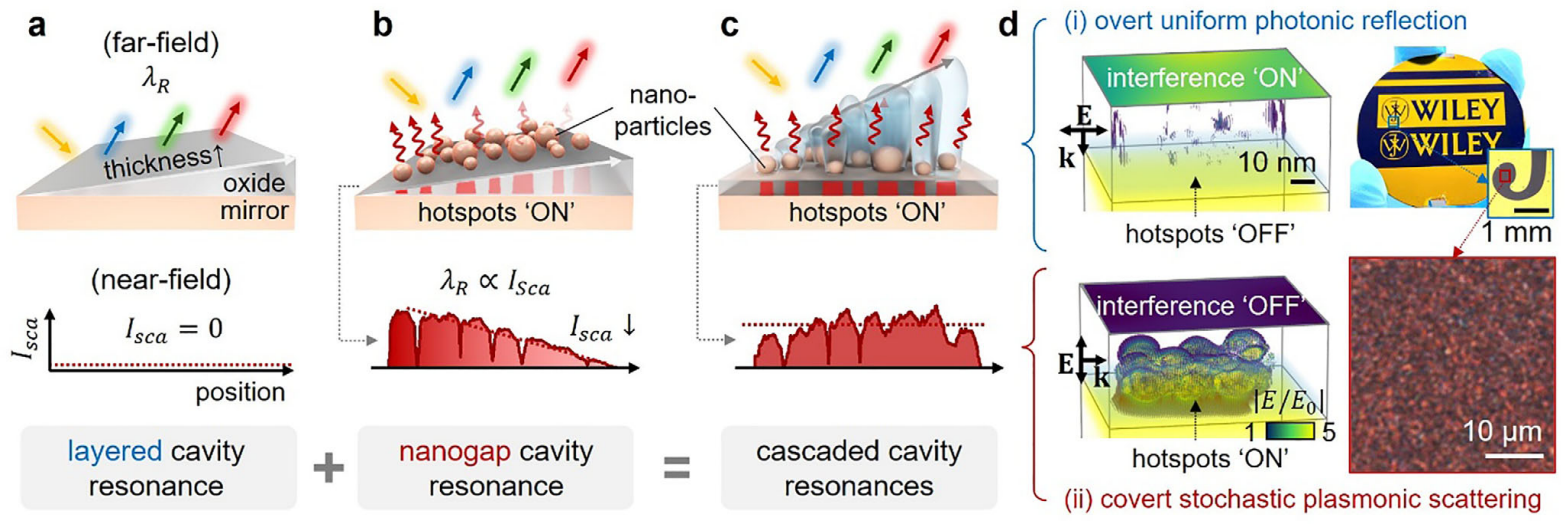
Concept of cascaded cavity resonances for overt–covert optical duality. (a) Layered cavity resonance producing deterministic far‐field photonic reflection through thickness‐dependent interference, with negligible stochastic scattering. (b) Nanogap cavity resonance based on stochastic nanoparticle assemblies, where localized plasmonic hotspots generate scattering responses that remain correlated with the reflection resonance. (c) Cascaded cavity resonances integrating layered and nanogap cavities, enabling overt uniform photonic reflection and covert stochastic plasmonic scattering with weak correlation between reflection wavelength (λ_*R*_) and scattering intensity (*I_sca_*). (d) Simulated optical near‐field distributions showing mode‐selective interference and hotspot activation in the cascaded cavity, together with experimental demonstration of macroscopic overt structural reflection and microscopic covert stochastic scattering fingerprints. The John Wiley & Sons, Inc. logo is reproduced with permission solely as a recognizable test pattern. It does not imply sponsorship or endorsement by publisher.

Nanophotonic structures have enabled precise manipulation of light through subwavelength optical resonance engineering [1, 2, 3, 4], leading to broad applications in structural coloration [5, 6, 7], displays [8, 9], sensing [10, 11, 12], and, more recently, optical information processing [13, 14, 15]. In particular, periodic and highly ordered photonic architectures, including photonic crystals [16], metasurfaces [17, 18], and layered cavities (e.g., Figure 1a) [19], have demonstrated deterministic optical functionalities governed by collective optical resonances such as coherent interference and diffraction. Such ordered nanophotonic systems provide highly uniform far‐field optical responses with controllable spectral characteristics, enabling vivid structural colors and engineered wavefront manipulation.

Beyond deterministic photonics, increasing attention has recently been directed toward disorder‐engineered optical systems [20, 21, 22, 23], where stochastic structural variations generate unconventional optical functionalities inaccessible in periodic architectures. Unlike ordered systems, disordered nanophotonic structures exhibit high optical entropy arising from nanoscale structural fluctuations, enabling random diffraction [24, 25], localized plasmonic coupling [14], and stochastic optical scattering [26]. These stochastic optical responses have attracted considerable interest for applications including physical unclonable functions (PUFs) [27], optical encryption [28], random lasing [29], and neuromorphic photonics [30], where unpredictability and irreproducibility are essential. However, achieving simultaneous control of overt optical appearance and covert stochastic optical information remains fundamentally challenging. In most existing disorder‐engineered photonic systems, both far‐ and near‐field optical responses originate from common optical resonances [31]. Consequently, enhancing optical randomness often degrades coherent structural colors, whereas improving photonic uniformity suppresses stochastic optical entropy. This intrinsic correlation limits the independent engineering of deterministic visual appearance and stochastic optical information within a single nanophotonic platform.

Recently, quasi‐ordered plasmonic metasurfaces demonstrated the coexistence of structural coloration and stochastic optical fingerprints through correlated nanoparticle assemblies (Figure 1b) [14]. However, because both the overt structural colors and covert stochastic scattering originated from the same nanogap cavity resonance, the stochastic scattering characteristics remained inherently correlated with the far‐field optical response. Alternatively, recent approaches have employed algorithm‐assisted entropy enhancement to generate distinct short‐ and long‐range optical interactions even from structurally correlated optical responses [32, 33]. Although these approaches improve statistical distinguishability, the generated optical entropy does not necessarily originate directly from independently engineered physical nanostructures. Therefore, a fundamentally new optical architecture capable of reducing the mutual dependence between deterministic far‐field colors from stochastic near‐field optical responses remains highly desirable.

Here, we introduce cascaded cavity resonances that enable covert optical fingerprints within overt structural colors through vertically integrated layered and nanogap cavities (Figure 1c). In this architecture, layered cavity resonances govern deterministic far‐field structural coloration, whereas localized nanogap cavity resonances generate stochastic optical scattering with minimal dependence on the overt optical appearance. By spatially separating these two optical channels across different cavity length scales, the proposed system overcomes the intrinsic trade‐off between optical uniformity and stochastic optical entropy. To realize this concept, we employ a monolithic vacuum‐based fabrication strategy that combines conventional thin film deposition with cooled glancing angle deposition, enabling densely packed yet short‐range correlated nanoparticle assemblies that intrinsically generate stochastic optical entropy. Furthermore, comprehensive reliability tests under mechanical, fluidic, and thermal stresses confirm the robust environmental stability of the cascaded structure, offering a scalable platform for integrating deterministic visualization with stochastic optical information within multifunctional nanophotonic systems.

## 2 Results and Discussion

### 2.1 Concept of Cascaded Cavity Resonance

Realizing overt structural colors and covert stochastic optical fingerprints within the same nanophotonic platform requires overcoming the intrinsic correlation between deterministic photonic resonances and disorder‐driven optical scattering. To address this challenge, we design a cascaded cavity architecture consisting of vertically integrated layered and nanogap cavities (Figure 1). As conceptually illustrated in Figure 1a, conventional layered cavity resonances generate deterministic far‐field photonic reflection through thickness‐dependent optical interference, producing highly uniform structural colors with negligible stochastic scattering. In contrast, nanogap cavity resonances formed by randomly distributed nanoparticles produce strongly localized plasmonic hotspots and stochastic scattering responses originating from nanoscale structural disorder (Figure 1b). However, because both the reflection resonance wavelength (λ_*R*_) and scattering intensity (*I_sca_*) are governed by the same plasmonic resonance channel, the overt optical appearance and covert stochastic scattering remain intrinsically correlated. This limitation can be overcome through cascaded cavity resonances, which vertically integrate layered photonic cavities and stochastic nanogap plasmonic cavities within a single monolithic architecture (Figure 1c). In this system, the layered cavity resonance governs far‐field uniform photonic reflection, while the nanogap cavity resonance generates near‐field stochastic plasmonic scattering with limited dependence on the reflection resonance wavelength. As experimentally demonstrated in Figure 1d, the resulting structure simultaneously exhibits highly uniform macroscopic structural blue colors and stochastic microscopic red scattering fingerprints, enabling weakly correlated engineering of overt optical appearance and covert optical information.

**FIGURE 2:**
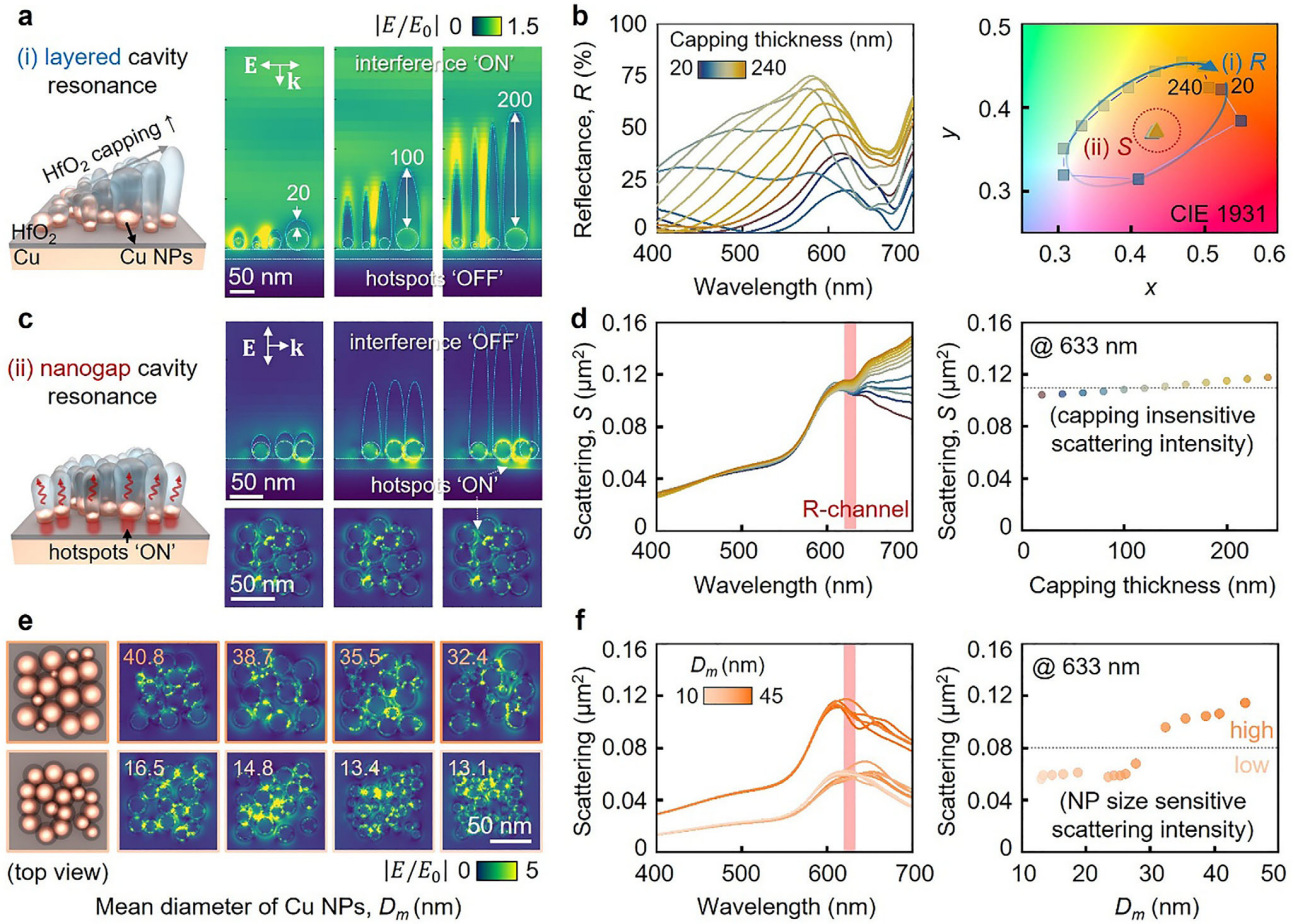
Theoretical analysis of cascaded cavity resonance. (a) Schematic illustration of the cascaded cavity for layered cavity resonances with varying HfO_2_ capping thicknesses (left) and corresponding optical near‐field distributions (right). (b) Simulated reflection spectra as a function of HfO_2_ capping thickness (left) and corresponding color distributions in the CIE 1931 chromaticity diagram (right). (c) Schematic illustration of the cascaded cavity for nanogap cavity resonances formed between Cu NPs and the Cu mirror (left) and corresponding localized optical near‐field enhancements within the dielectric nanogap region (right). (d) Simulated scattering spectra for different HfO_2_ capping thicknesses (left) and associated scattering intensity at λ = 633 nm (right). (e) Simulated optical near‐field distributions for different mean diameters of Cu NPs at a constant surface coverage of 49.5% within a simulation area of 120 × 120 nm^2^. (f) Associated simulated scattering spectra (left) and scattering intensity at λ = 633 nm (right).

To investigate the physical origin of this optically weak coupling, we perform finite‐difference time‐domain (FDTD) simulations for randomly distributed Cu nanoparticles (Cu NPs, average diameter: 16.5 nm) positioned above a Cu mirror separated by a 20 nm HfO_2_ dielectric gap layer (Figure 2a). Cu is selected as the plasmonic material because it exhibits optical properties comparable to Au in the visible regime [34], while offering substantially lower material cost and compatibility with large‐scale manufacturing [35]. Despite these advantages, Cu NPs generally suffer from rapid oxidation and aggregation, which limit their stability and practical implementation in plasmonic systems [36]. To address this issue, HfO_2_ is employed as the dielectric material due to its high refractive index and excellent chemical stability, enabling efficient layered cavity resonances while simultaneously encapsulating and protecting the Cu NPs [37]. The key variable is the thickness of the upper HfO_2_ capping layer, which systematically varies from 20 to 240 nm to analyze the resulting reflection spectra and optical near‐field distributions (Figure S1). By calculating the effective refractive index of the mixed layer composed of Cu NPs and HfO_2_, the overall structure can be approximated as a multilayered cavity system. Consequently, increasing the HfO_2_ capping thickness increases the optical path length within the layered cavity, thereby continuously shifting the interference condition and resonance wavelength. As shown in the left panel of Figure 2b, varying the HfO_2_ capping thickness strongly modifies the far‐field reflection spectrum while only weakly perturbing the localized scattering response. This interpretation is further supported by the CIE chromaticity analysis in the right panel of Figure 2b and the CIEDE2000 color‐distance analysis of the simulated spectra in Figure S2 and Table S1. Excluding the 20 nm reference condition, the simulated scattering and reflection channels exhibit mean *ΔE* values of 0.736 and 28.58, respectively. The mean color difference of the scattering channel is below the commonly reported just‐noticeable difference of approximately 1, whereas that of the reflection channel indicates readily distinguishable color modulation. These results demonstrate that the HfO_2_ capping layer predominantly controls the overt reflection color while producing only limited perceptual variation in the simulated scattering color. These results confirm that the overt optical appearance is predominantly governed by the layered cavity resonance.

In contrast, covert stochastic optical information originates from nanogap cavity resonances formed between the Cu NPs and the Cu mirror. To assess the degree of correlation between the overt structural colors and covert stochastic scattering, we analyze the scattering response over the same HfO_2_ capping thickness range (20–240 nm). Interestingly, despite substantial modulation of the reflection spectra, the optical near‐field remains strongly localized within the HfO_2_ nanogap region for all capping thicknesses, exhibiting pronounced plasmonic hotspots with minimal variation in near‐field intensity (Figure 2c and Figure S3). Correspondingly, the scattering spectra show only minor variations throughout the thickness range in the visible regime (Figure 2d). In particular, the scattering intensity at 633 nm, corresponding to the representative red‐channel wavelength in the imaging system, changes only from 0.104 to 0.117 µm^2^ as the capping thickness increases from 20 to 240 nm, corresponding to a total variation of merely 12.5% (right panel of Figure 2d). A Pearson correlation analysis between the reflection peak wavelength and the scattering intensity at 633 nm yields coefficients of 0.34 for the simulation (0.53 for the experiment, bottom panel of Figure S2), indicating a finite correlation rather than strict statistical independence. Nevertheless, the substantially smaller modulation of the scattering response compared with the reflection channel demonstrates that the two optical functionalities remain only weakly coupled to the HfO_2_ capping thickness. These results therefore support a weakly correlated functional relationship between the overt photonic reflection and covert stochastic scattering channels, allowing substantial tuning of the former with comparatively limited perturbation of the latter.

Crucially, while the far‐field coloration is regulated by the capping thickness, the stochastic scattering response can be systematically engineered through the nanoparticle geometry (Figure 2e). Specifically, we systematically vary the average diameter of the Cu NPs (*D_m_*) from 13.1 to 44.8 nm while maintaining a constant surface coverage of 49.5%, consistent with the experimentally observed structural evolution discussed in the following section. Increasing the mean diameter of Cu NPs significantly modifies the optical near‐field confinement within the nanogap cavity, thereby strongly altering the local scattering spectra calculated over a fixed area (120 × 120 nm^2^, corresponding to the nominal object‐plane area of one 2 × 2 binned pixel used in the scattering measurements described below). In particular, the scattering intensity at 633 nm increases from 0.05 to 0.12 µm^2^ with increasing mean diameter, corresponding to more than a twofold enhancement (Figure 2f). This pronounced dependence originates from size‐dependent localized plasmonic confinement within the nanogap cavity [8], providing a physically engineered origin for stochastic optical entropy generation. Therefore, the proposed cascaded cavity architecture enables weakly correlated engineering of deterministic overt structural colors and covert stochastic optical fingerprints within a single monolithic nanophotonic platform.

### 2.2 Monolithic Growth of Short‐Range Correlated Cascaded Cavities

**FIGURE 3:**
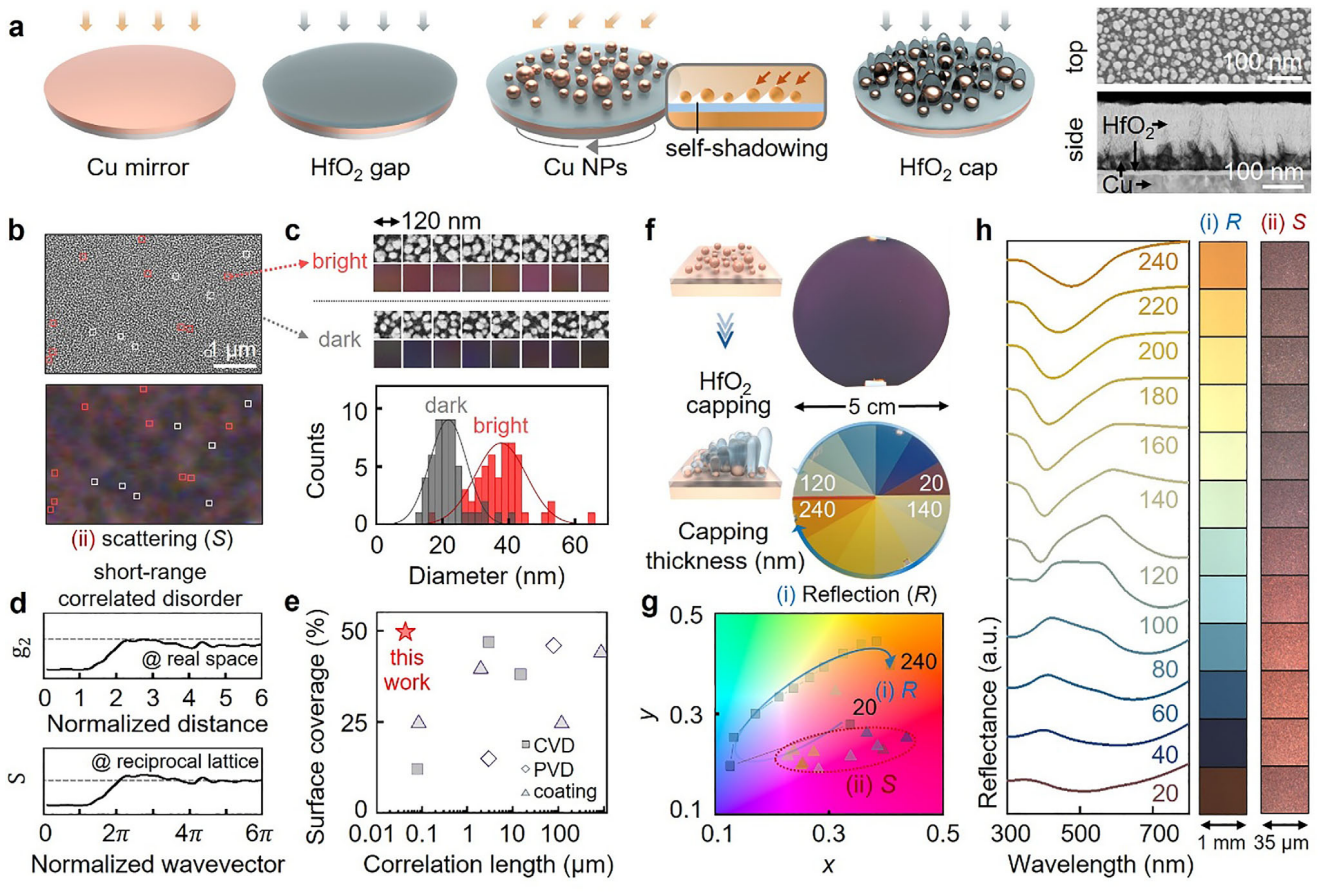
Monolithic growth of short‐range correlated cascaded cavities and their optical characteristics. (a) Monolithic physical vapor deposition process for fabrication of the cascaded cavity (right: electron microscopy images of the fabricated cascaded cavity). (b) Corresponding SEM image and stochastic optical scattering image of the cascaded cavity. (c) Representative bright (red box) and dark (white box) scattering regions randomly selected from the same sample area (120 × 120 nm^2^). (d) Structural correlation analysis of the fabricated Cu NP assemblies in real space using the pair correlation function *g_2_* (top) and in reciprocal space using the structure factor *S* (bottom). (e) Correlation length and nanoparticle surface coverage of the proposed cascaded cavity compared with representative disorder‐engineered optical surfaces reported in previous studies [14, 41, 42, 43, 44, 45, 46, 47, 48]. (f) Schematic illustration (left) and digital photographs (right) of cascaded cavities exhibiting different overt structural colors through variation of the HfO_2_ capping thickness. (g) Corresponding color distributions in the CIE 1931 chromaticity diagram, and (h) reflection spectra (left), far‐field reflection images (middle), and covert stochastic scattering images (right).

The cascaded cavity architecture is fabricated entirely through a monolithic physical vapor deposition process using only Cu and HfO_2_ (Figure 3a), both of which are widely adopted in semiconductor manufacturing owing to their scalability and process compatibility [38]. While the Cu mirror and both top and bottom HfO_2_ dielectric layers are sequentially deposited using conventional deposition to form the layered cavity structure, we grow a monolayer of disordered Cu NPs by glancing angle deposition (GLAD) with substrate cooling [2]. By tilting the substrate to approximately 85° relative to the incident vapor flux together with azimuthal rotation, self‐shadowing during the early nucleation stage suppresses continuous film growth and instead promotes spontaneous formation of spatially disordered three‐dimensional Cu NPs [39]. Simultaneously, substrate cooling kinetically suppresses adatom surface diffusion, thereby preventing excessive nanoparticle aggregation and coalescence during growth (Figure 3b) [40]. As a result, the cooled GLAD process enables highly dense yet positionally short‐range nanoparticle assemblies with high spatial uniformity over large areas. Subsequently, we deposit an additional HfO_2_ capping layer to complete the vertically integrated cascaded cavity architecture, which further governs deterministic far‐field photonic reflection and structural coloration (e.g., Figure 3f). Because the entire fabrication process relies exclusively on vacuum‐based deposition, the proposed platform is inherently scalable and compatible with semiconductor manufacturing.

We then experimentally investigate the structural origin of covert stochastic scattering by correlating local configurations of Cu NPs with the corresponding optical scattering responses obtained from randomly selected 120 × 120 nm^2^ regions within the same dark‐field scattering image (Figure 3b,c). Representative bright and dark scattering domains are chosen as the regions with the highest and lowest average red‐channel intensities among the selected regions, respectively. Electron microscopy analysis reveals clear differences in local Cu NP geometry depending on the scattering intensity. Specifically, bright scattering regions contain substantially larger nanoparticles than dark regions, with average nanoparticle diameters of 37.7 ± 6.4 nm and 21.9 ± 4.1 nm, respectively (Figure 3c). These results indicate that stochastic scattering intensity is strongly governed by local nanoparticle geometry within the nanogap cavity and provides a direct physical link between local nanoparticle geometry and stochastic optical information generation.

We further quantitatively analyze the spatial arrangement of the Cu NPs using the pair correlation function *g*_2_ in real space and the structure factor *S* in reciprocal space (Figure 3d and Figure S4, see Note S1 for detail). The pair correlation analysis shows that neighboring Cu NPs exhibit only short‐range positional correlation and rapidly lose spatial correlation beyond several tens of nanometers. Importantly, no long‐range ordering or large‐scale aggregation is observed despite the high nanoparticle density. Consistently, the structure factor analysis reveals suppressed long‐wavelength density fluctuations without periodic ordering, confirming that the Cu NPs form a densely packed yet short‐range correlated disordered structure rather than clustered or periodic nanoparticle networks. This unusual behavior originates from kinetically frozen disorder during cooled GLAD growth, where self‐shadowing promotes dense nanoparticle nucleation while suppressed adatom diffusion prevents excessive nanoparticle coalescence. Consequently, the growth process simultaneously achieves high nanoparticle surface coverage (49.5%) and an extremely short correlation length of 44 nm (Figure 3e and Table S2). This short structural correlation length promotes nanoscale configurational variability, thereby providing a favorable structural basis for high‐density stochastic optical encoding. Compared with representative disorder‐engineered optical surfaces fabricated using previous approaches [14, 41, 42, 43, 44, 45, 46, 47, 48], our cascaded cavity exhibits substantially shorter correlation lengths despite the high nanoparticle density, successfully breaking the conventional trade‐off between structural density and spatial randomness. Such densely packed yet short‐range correlated nanoparticle assemblies enhance local stochastic variability while supporting high spatial encoding density, providing a major advantage for high‐density stochastic optical information encoding.

Another key advantage of our cascaded cavity architecture is that overt structural colors can be engineered by simply varying the HfO_2_ capping thickness after Cu NP growth (Figure 3f and Figure S5). As the capping thickness increases from 20 to 240 nm, the far‐field reflection color continuously evolves across the visible regime owing to modulation of the layered cavity resonance condition. The measured reflection spectra exhibit systematic spectral shifts consistent with the corresponding CIE 1931 chromaticity coordinates (Figure 3g,h), confirming deterministic tuning of overt structural colors through the layered photonic cavity. Importantly, despite these substantial changes in macroscopic optical appearance, the covert stochastic scattering patterns remain consistently preserved throughout the entire thickness range. This behavior directly verifies that the stochastic plasmonic scattering response is largely insensitive to the layered cavity resonance and instead governed primarily by localized nanogap cavity interactions. The control structures further reveal the distinct roles of the two constituent cavities (Figure S6), i.e., the layered cavity provides tunable far‐field reflection colors with negligible scattering, whereas the nanogap cavity generates stochastic scattering while maintaining an essentially fixed reflection color. Consequently, our cascaded cavity resonances experimentally demonstrate weak optical correlation between overt photonic reflection and covert stochastic optical fingerprints, enabling weakly correlated yet effectively tunable structural colors and stochastic optical information within a single monolithic nanophotonic platform.

### 2.3 Covert Optical Fingerprint From Cascaded Cavities

**FIGURE 4:**
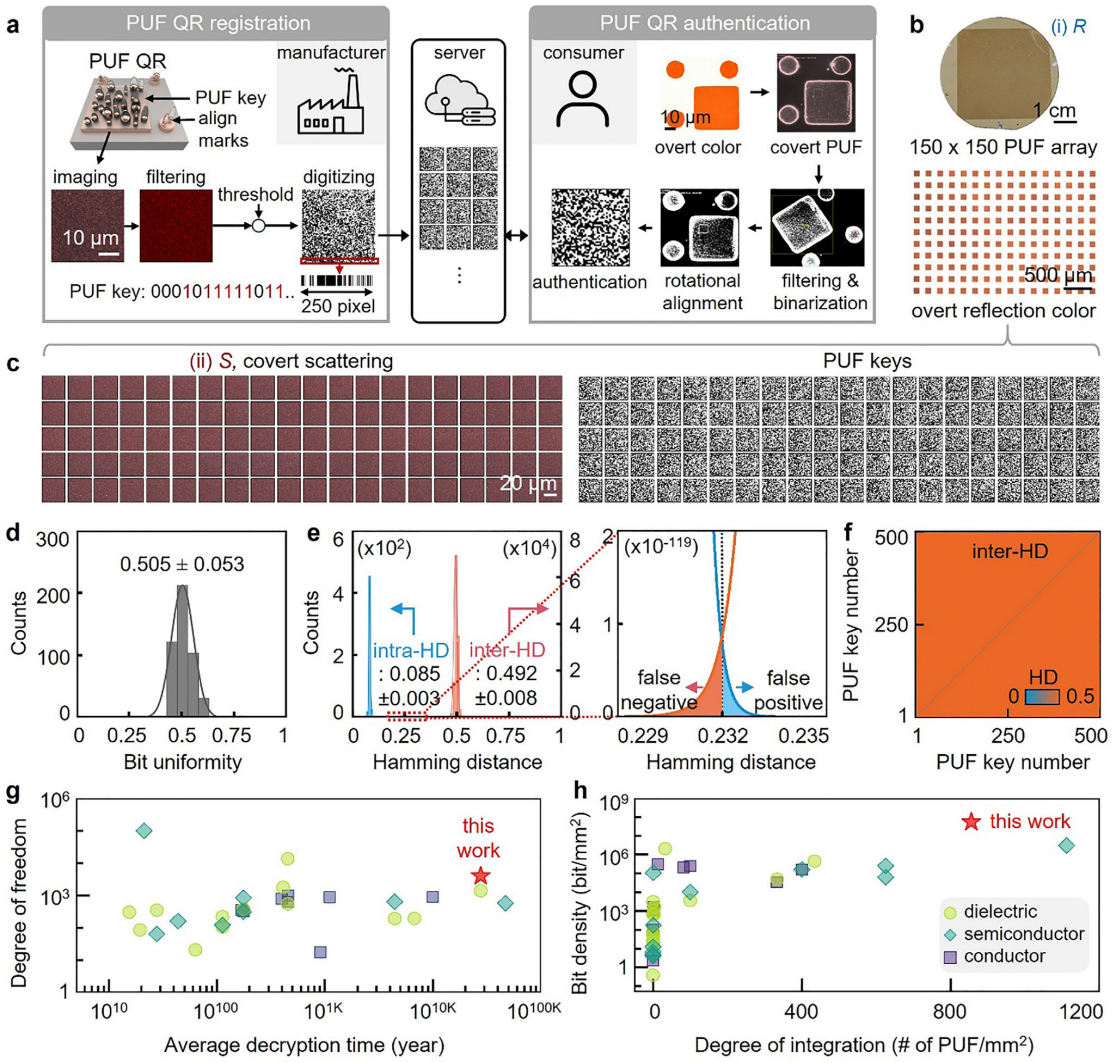
Performance of PUF keys. (a) Schematic illustration of registration and authentication process of PUF QR keys. (b) Fabricated cascaded cavities on a 2‐inch wafer (each area: 100 × 100 µm) (top), and randomly selected 500 PUF keys' reflection (bottom), (c) scattering (left), and generated PUF keys (right). (d) Corresponding 500 PUF keys' bit uniformity, (e) inter‐ and intra‐Hamming distance (left), Gaussian fits‐based estimates of FNR and FPR (right), and (f) 2D pair inter‐Hamming distances. Comparison of (g) average physical decryption times with its degree of freedom (with 29 literatures) and (h) degree of integration with bit density (with 44 literatures).

The covert stochastic optical information generated from the cascaded cavity can be directly utilized as a physical unclonable function for anti‐counterfeiting and product authentication (Figure 4). Unlike conventional stochastic optical PUFs that often require precise positioning during optical readout, we integrate overt optical alignment marks together with covert stochastic fingerprints, enabling alignment‐assisted registration and authentication processes (Figure 4a and Figure S7, Video S1). In an authentication workflow, stochastic scattering patterns are first converted into digitized binary fingerprints through image filtering and thresholding and subsequently stored in a secure database. During authentication, the overt alignment marks enable rotational and positional correction prior to stochastic fingerprint extraction, thereby allowing rapid and reliable machine‐readable authentication. This alignment‐assisted architecture significantly improves practical usability for large‐area and high‐throughput optical security applications.

We fabricate such 22,500 PUF arrays (150 × 150 cascaded cavities) on a single 2‐inch wafer (Figure 4b). Importantly, all devices exhibit nearly identical overt reflection colors across the wafer scale, confirming the robustness of the layered cavity resonance together with excellent process uniformity over large areas. In contrast, each device simultaneously generates distinct covert stochastic scattering patterns owing to the random Cu NP distribution. When digitized through image binarization, every scattering pattern produces an individual optical fingerprint with high stochastic distinguishability (Figure 4c and Figure S8). To statistically investigate the stochastic information characteristics enabled by the cascaded cavity architecture, we randomly select 500 PUFs from the fabricated wafer and analyze them in terms of bit uniformity (Figure 4d), inter‐/intra‐Hamming distance (HD, left panel of Figure 4e), authentication error probability (right panel of Figure 4e). All generated bit streams successfully pass the NIST statistical randomness tests (Table S3). The measured bit uniformity is 0.505 ± 0.053, closely matching the ideal value of 0.5 and indicating highly balanced stochastic entropy generation. Pairwise distinguishability between independently generated PUFs yields an inter‐HD of 0.492 ± 0.008, remarkably close to the ideal value of 0.5, confirming near‐ideal pairwise distinguishability between different stochastic optical fingerprints. To visually confirm this wafer‐scale uniqueness, the pairwise inter‐HD values are mapped onto a 2D spatial matrix (Figure 4f), which demonstrates a highly homogeneous distribution of randomness across the entire wafer surface without an obvious wafer‐scale regional dependence. In contrast, the intra‐HD, representing the reproducibility of repeatedly measured identical PUFs, is 0.085 ± 0.003. While slightly deviating from the ideal value of zero, this marginal intra‐HD increment represents a deliberate engineering trade‐off. A 2 × 2 pixel‐binning condition is selected as the smallest practical encoding unit that preserves strong stochastic contrast while limiting excessive spatial averaging (Figure S9). Because neighboring optical pixels may remain correlated owing to the finite imaging resolution, we additionally evaluate the spatial autocorrelation of the raw red‐channel scattering images obtained from 500 PUFs (Note S2). The normalized autocorrelation decays to 1/*e* at an average radial distance of 3.67 raw pixels, corresponding to approximately 220 nm in the object plane. This correlation length is larger than the nominal 120 nm pitch of one 2 × 2 binned encoding pixel, indicating that adjacent encoded pixels are not fully statistically independent. Accordingly, the pixel‐based bit density reported here is referred to as the nominal encoding density, whereas the measured autocorrelation provides an estimate of the effective spatial degrees of freedom. Nevertheless, the proposed encoding condition provides a practical balance between high spatial information density and reliable authentication reproducibility. Gaussian fitting of the intra‐ and inter‐HD distributions reveals an optimal authentication threshold at 0.232 (right panel of Figure 4e) [49]. Based on this statistically fitted threshold, Gaussian extrapolation of the fitted distributions yields estimated false negative rate (FNR) and false positive rate (FPR) probabilities of 3.1 × 10^−^^124^ and 8.4 × 10^−^^120^, respectively. These values represent extrapolated probabilities beyond the experimentally sampled range rather than directly measured error rates. Nevertheless, within the Gaussian model, they indicate statistically negligible authentication failure probabilities.

To further benchmark the stochastic encoding capability, the proposed system is compared with previously reported optical PUF platforms in terms of practical key capacity and resistance against random guessing attacks (Figure 4g). The effective statistical degree of freedom estimated from the inter‐PUF response distribution reaches 2^3,905^ (average decryption time ∼3.3 × 10^27,686^ years), substantially exceeding previous optical PUF systems. In addition to stochastic distinguishability, our platform exhibits exceptional physical integration density. The degree of integration and nominal encoding density reach 854.9 PUF/mm^2^ and 6.94 × 10^7^ bit/mm^2^, respectively, both surpassing previously reported optical PUF platforms (Figure 4h, see Table S4 for details) [14, 32, 42, 43, 44, 45, 46, 47, 48, 50, 51, 52, 53, 54, 55, 56, 57, 58, 59, 60, 61, 62, 63, 64, 65, 66, 67, 68, 69, 70, 71, 72, 73, 74, 75, 76, 77, 78, 79, 80, 81, 82, 83, 84, 85, 86, 87]. This advantage originates from the densely packed yet weakly correlated Cu NP assemblies, which simultaneously maintain a high nanoparticle surface coverage (49.5%) and a short correlation length (∼44 nm). As a result, stochastic optical fingerprints can be encoded within highly confined spatial regions without sacrificing distinguishability. Crucially, while such a highly disordered and dense nanoparticle network would inevitably disrupt macroscopic visual quality in conventional single‐resonance security media, our cascaded cavity architecture effectively circumvents this constraint. These results demonstrate that cascaded cavity resonances enable simultaneous optimization of stochastic encoding capacity and visual uniformity, providing a scalable framework for integrating overt structural colors with covert optical information.

### 2.4 Application and Environmental Robustness of Cascaded Cavities

**FIGURE 5:**
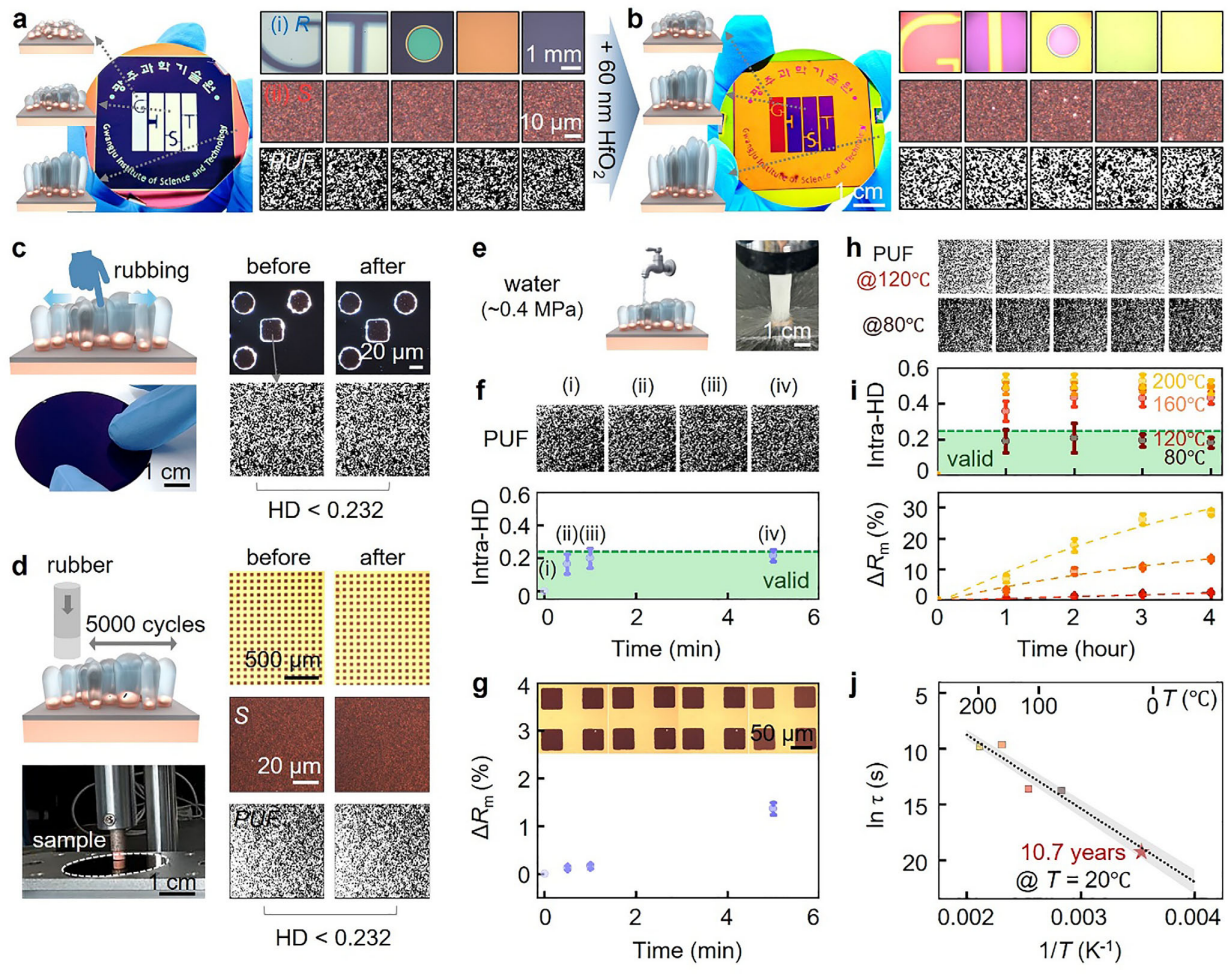
Programmable overt structural colors and environmental robustness of cascaded cavities. (a) Cascaded cavities exhibiting different overt structural colors with corresponding covert stochastic scattering patterns generated by varying the HfO_2_ capping thickness. (b) Extended structural color library achieved by additionally depositing a uniform 60 nm‐thick HfO_2_ layer while preserving stochastic scattering characteristics. The university logo is reproduced under institute's permission. (c) Manual finger‐rubbing test and corresponding scattering images and PUF keys before and after rubbing. (d) Controlled abrasion test for 5000 cycles and corresponding reflection, scattering, and PUF responses. (e–g) High‐pressure water‐exposure test (∼0.4 MPa), regenerated PUF responses, and average reflectance variation. (h, i) Regenerated PUF keys, intra‐Hamming distances, and reflectance variations during accelerated thermal aging at 80°C–200°C. (j) Estimated operational lifetime at 20°C obtained by Arrhenius extrapolation (shaded region: 95% confidence interval of the Arrhenius fit). For the quantitative robustness tests, abrasion was evaluated at 15 spatially separated PUF locations on one sample; water exposure was evaluated repeatedly at the same five locations on one sample; and thermal aging used a separate sample for each temperature, with the same five locations tracked over time. Error bars represent the standard deviation across the measured locations.

The cascaded cavity simultaneously combines overt structural color engineering with covert stochastic optical fingerprinting, enabling covert optical information to be embedded within visually recognizable macroscopic patterns. To demonstrate this capability, we fabricate a multicolored university logo on a 2‐inch wafer using a shadow mask‐assisted monolithic deposition process (left panel of Figure 5a and Figure S10). During fabrication, the Cu mirror, HfO_2_ gap layer, and Cu NP layer are uniformly deposited across the entire wafer, while only the upper HfO_2_ capping layer is selectively patterned through sequential shadow mask deposition. By varying the HfO_2_ thicknesses (20, 80, and 100 nm), the layered cavity resonance continuously evolves, producing visually distinguishable structural colors including green, yellow, orange, and purple. Importantly, although the overt reflection colors change significantly across the patterned regions, the covert stochastic scattering patterns remain nearly unchanged throughout the entire university logo structure, confirming weak correlation between overt photonic reflection and covert stochastic optical fingerprints (right panel of Figure 5a). We further achieve inverted‐tone structural color contrast by additionally depositing a uniform 60 nm‐thick HfO_2_ layer across the wafer, which selectively modulates the upper layered cavity interference while leaving the underlying nanogap geometries intact, thereby maintaining nearly identical stochastic scattering characteristics (Figure 5b). These results demonstrate that overt structural colors can be continuously engineered without disrupting covert stochastic optical information generation.

We next investigate the practical robustness of the cascaded cavity platform under representative environmental and mechanical stress conditions including mechanical abrasion (Figure 5c,d), water exposure (Figure 5e–g and Figure S11), thermal annealing (Figure 5h,i and Figure S12), and estimation of long‐term aging (Figure 5j). Mechanical durability is first evaluated through both manual finger‐rubbing tests (Figure 5c and Video S2) and repeated abrasion tests up to 5000 cycles (Figure 5d and Video S3). In both cases, the overt structural colors, stochastic scattering images, and corresponding digitized optical fingerprints remain well preserved after repeated mechanical deformation. Quantitatively, the regenerated optical fingerprints consistently maintain Hamming distances below the authentication threshold of 0.232 even after 5000 abrasion cycles, while the overt reflection color pixel value changed by less than 3.5%. These results indicate that the cascaded cavity preserves both overt and covert optical functionalities under severe mechanical stress.

Beyond dry mechanical deformation, we expose the cascaded cavity to high‐pressure water flow (∼0.4 MPa), corresponding to harsh aqueous environments such as washing or heavy rainfall (Figure 5e and Video S4). Even after continuous water exposure for up to 5 min, the intra‐HDs of the regenerated stochastic optical fingerprints remain below the authentication threshold, confirming preservation of stochastic optical distinguishability under liquid‐phase environments (Figure 5f). Simultaneously, the overt structural colors exhibit only ∼1.5% average reflectance variation across the visible wavelength range (400–700 nm) after water exposure (Figure 5g). Because liquid‐phase water exposure imposes substantially harsher conditions than gas‐phase humidity, these results strongly suggest excellent environmental stability of both overt and covert optical functionalities.

We further investigate the thermal and temporal robustness of the cascaded cavity using accelerated aging analysis based on an Arrhenius model under different thermal exposure conditions (Figure 5h and Note S3) [88]. Individual cascaded cavity samples are thermally annealed at 80°C, 120°C, 160°C, and 200°C for durations up to 4 h, followed by regeneration of the corresponding stochastic optical fingerprints through re‐imaging. Up to 120°C, all regenerated fingerprints maintain HDs below the authentication threshold of 0.232 (top panel of Figure 5i), indicating stable stochastic optical operation under elevated temperatures relevant to practical outdoor and industrial environments. In contrast, accelerated degradation becomes observable above 160°C, where both reflectance modulation and stochastic fingerprint degradation progressively increase with annealing duration. To quantitatively estimate the long‐term operational stability, we analyze the average reflectance variation as a function of annealing temperature and time. The reflectance degradation follows exponential decay behavior, enabling extraction of characteristic decay constants through exponential fitting (bottom panel of Figure 5i). Arrhenius extrapolation of the extracted decay rates yields an estimated operational lifetime of approximately 10.7 years at 20°C (Figure 5j), indicating excellent long‐term stability of the monolithically integrated cascaded cavity platform. Collectively, these results demonstrate that cascaded cavity resonances enable programmable overt structural coloration and robust covert stochastic optical information within a mechanically, environmentally, and thermally resilient nanophotonic platform.

## 3 Conclusions

We demonstrate cascaded cavity resonances that weakly correlate deterministic photonic reflection with stochastic plasmonic scattering within a single monolithic nanophotonic architecture. By vertically integrating layered photonic cavities and localized plasmonic nanogap cavities, the proposed system enables effectively tunable overt structural colors and covert optical fingerprints through weakly correlated optical channels across different optical length scales. Consequently, far‐field structural coloration can be continuously programmed without sacrificing the stochastic optical information required for authentication and encoding. This overt–covert optical duality overcomes a longstanding trade‐off between optical uniformity and optical randomness, establishing a new strategy for integrating deterministic and stochastic photonic functionalities within the same platform.

A key aspect of this architecture lies in the combination of monolithic vacuum‐based fabrication and physically engineered disorder. By coupling conventional thin‐film deposition with cooled glancing‐angle deposition, we realize densely packed yet weakly correlated Cu nanoparticle assemblies that simultaneously maintain high surface coverage and short correlation lengths. Such disorder enables spatially compressed stochastic optical entropy while preserving wafer‐scale manufacturability, thereby bridging a gap that often exists between nanophotonic discovery and practical implementation. The resulting platform supports large‐scale optical fingerprint generation together with robust operation under mechanical, aqueous, and thermal environments.

Beyond optical PUFs, cascaded cavity resonances provide a general framework for integrating visible and hidden optical functionalities within the same nanophotonic system. More broadly, the concept of weakly correlated multiscale cavity resonances introduces a new design paradigm in which deterministic optical responses and stochastic optical information can be engineered with limited mutual influence while coexisting within a common material platform. We anticipate that this strategy will inspire future multifunctional photonic systems that integrate visualization [10, 11, 89, 90], information encoding [13, 14, 91], and adaptive optical functionalities [92, 93], within a single scalable architecture.

## 4 Methods

### 4.1 Numerical Simulation

The optical properties of the cascaded cavity architecture were calculated using a commercial finite‐difference time‐domain (FDTD) simulation package (Ansys Lumerical, 2024 R2). The lateral simulation domain was set to 120 × 120 nm^2^, corresponding to the nominal object‐plane area represented by one 2 × 2 binned pixel used for experimental PUF encoding (Figure S13). This simulation domain represents a local stochastic sampling area corresponding to the experimental encoding grid and should not be interpreted as the diffraction‐limited optical resolution or as a fully independent optical sampling area. The Cu nanoparticles were modeled as non‐overlapping spheres with stochastic lateral positions. For each simulated mean‐particle‐size condition, individual nanoparticle diameters were distributed around the prescribed mean diameter, thereby representing the size dispersion of the experimentally formed nanoparticles. The number and diameters of the nanoparticles were adjusted while maintaining a constant surface coverage of approximately 49.5%. Consequently, the number of nanoparticles within the simulation domain varied with the prescribed mean particle diameter. Particle overlap was prohibited during stochastic structure generation. A total of 10 mean‐particle‐size conditions were modeled to investigate the effect of nanoparticle size at constant surface coverage. For simulations examining the effect of HfO_2_ capping thickness, the same nanoparticle geometry and spatial configuration were retained for all thickness conditions, with only the HfO_2_ thickness varied. This approach isolates the thickness‐dependent optical response from variations associated with different nanoparticle configurations. Perfectly matched layer (PML) boundary conditions were applied along the *x*, *y*, and *z* directions to suppress artificial reflections from the finite simulation boundaries. A uniform mesh size of 1 nm was used along all three spatial directions throughout the simulation domain. A normally incident plane wave was illuminated toward the cascaded layers to calculate the reflectance spectra. Reflectance spectra were obtained over the wavelength range of 400 – 800 nm with a spectral interval of 1 nm. The optical constants of HfO_2_ and Cu were obtained from established literature [94, 95].

### 4.2 Growth of Thin Films

A 150 nm‐thick Cu film and a 20 nm‐thick HfO_2_ layer were sequentially deposited on a 2‐inch silicon wafer by electron‐beam evaporation at a deposition rate of 0.1 nm/s. During conventional thin‐film deposition, the substrate was cooled to −35°C and azimuthally rotated at 300 rph. To spontaneously form spatially disordered Cu NP monolayers, cooled glancing‐angle deposition was performed by tilting the substrate to 85° relative to the incident vapor flux while reducing the azimuthal rotation speed to 10 rotations/h. The nominal deposition thickness monitored by a quartz crystal microbalance (QCM) was 300 nm. Subsequently, an additional HfO_2_ capping layer with thicknesses ranging from 20 to 240 nm was deposited onto the Cu NPs by electron‐beam evaporation at a deposition rate of 0.1 nm/s under substrate cooling. For patterning the HfO_2_ capping layer to integrate alignment marks and QR‐like structures, a photoresist (AZ‐5214, Sigma‐Aldrich) was spin‐coated onto the fabricated substrate at 4000 rpm for 35 s and soft‐baked at 110°C for 90 s. The photoresist was then exposed to UV light using a mask aligner (M100, Prowin; wavelength: 400 nm; power density: 20 mW/cm^2^) for 7 s and developed in 300 MIF developer (Sigma‐Aldrich) for 30 s.

### 4.3 Electron Microscope Analysis

The structural characteristics of the cascaded cavities were examined using scanning electron microscopy (SEM; SU5000, Hitachi) and transmission electron microscopy (TEM; Tecnai G2 F30 S‐Twin, FEI). For surface SEM analysis, an accelerating voltage of 15 kV was used. Morphological images were acquired from three randomly selected regions with an area of 2.1×1.4 µm^2^ per sample to statistically evaluate the surface coverage and local geometry of the Cu NPs. Cross‐sectional structural characterization was performed using TEM operated at an accelerating voltage of 300 kV. Prior to TEM imaging, cross‐sectional specimens were prepared using a focused ion beam system (FIB; NX5000, Hitachi).

### 4.4 Optical Imaging and Spectroscopy

Bright‐field reflection and dark‐field scattering images and spectra of the cascaded cavity platforms were obtained using a customized microscope setup (BX53, Olympus) equipped with a CMOS camera (STC‐MCS500U3V, Sentech) and a spectrometer (QE Pro, Ocean Insight). Optical measurements were performed using a 5× objective lens (MPLFLN‐BD, Olympus; numerical aperture: 0.15) for large‐area macroscopic imaging and a 100× objective lens (MPLFLN‐BD, Olympus; numerical aperture: 0.90) for high‐resolution microscopic characterization. A halogen lamp (7724, PHILIPS; power: 100 W) was used as the white‐light source. To select the representative bright and dark scattering regions shown in Figure 3c, more than 20 local regions (120 × 120 nm^2^) were randomly selected from the same dark‐field scattering image. The average red‐channel intensity of each region was quantitatively evaluated, and the regions exhibiting the highest and lowest average red‐channel intensities were defined as the representative bright and dark scattering regions, respectively. The corresponding locations were then identified in the SEM image, and the local Cu NP size distributions were analyzed to correlate the nanoparticle geometry with the measured stochastic scattering intensity.

### 4.5 Generation of PUF Keys

Microscopic optical scattering images obtained from the cascaded cavities were separated into red, green, and blue color channels. The red‐channel images were selectively used for PUF key generation. Each image was cropped to 500 × 500 pixels and spatially binned to 250 × 250 pixels to optimize entropy extraction and readout reproducibility. To convert the analog optical scattering images into binary bit streams, a grayscale threshold of 120 was applied. Pixels with intensities below 120 were decoded as response bits of “0” corresponding to dark domains, whereas pixels with intensities above 120 were decoded as response bits of “1” corresponding to bright domains.

### 4.6 Mechanical Robustness Test

The mechanical durability of the cascaded cavity platform was evaluated using a one‐axis abrasion tester (Daesung Precision, South Korea) equipped with a rubber contact probe. A cascaded cavity sample was securely mounted on a stage, and repeated linear rubbing was applied under a normal pressure of 9.4 MPa for 5000 cycles. Fifteen spatially separated PUF patterns on the same sample were characterized before and after abrasion, and the preservation of the overt structural color and reproducibility of the regenerated PUF keys were quantitatively evaluated.

### 4.7 High‐Pressure Water Test

To simulate harsh fluidic environments, a cascaded cavity sample was exposed to continuous high‐pressure water spray at approximately 0.4 MPa for controlled durations of 30, 60, and 300 s. Five spatially separated PUF patterns on the same sample were selected, and the same five locations were repeatedly characterized throughout the exposure test. After each exposure duration, PUF fingerprints and reflection spectra were acquired from the same five locations to evaluate the reproducibility of the covert and overt optical responses.

### 4.8 Aging Acceleration Test

Accelerated aging analysis of the monolithically integrated cascaded cavities was performed based on the Arrhenius relationship to estimate the long‐term thermal stability and operational lifetime of the optical PUF platform. Separate physical samples were used for each annealing temperature (80°C, 120°C, 160°C, and 200°C). For each temperature‐specific sample, five spatially separated PUF locations were selected and the same locations were repeatedly characterized at 1 h intervals over an annealing duration of 0–4 h. After each thermal stress step, the optical properties and intra‐Hamming distances of the regenerated PUF keys were monitored. Simultaneously, the average reflectance variation within the visible wavelength range of 400–700 nm was analyzed as a function of annealing duration for each temperature condition. The temporal reflectance degradation followed exponential relaxation behavior, enabling extraction of characteristic decay time constants through exponential fitting. To predict the device operational lifetime under ambient conditions at 20°C, the extracted time constants were plotted against inverse absolute temperature according to the Arrhenius relationship and analyzed by linear fitting. The activation energy of the cascaded cavity was calculated to be 0.568 eV.

## Author Contributions

G.K., Y.M.S., and H.‐H.J. designed experiments. G.K. and J.L. performed the simulation. G.K. and J.M. performed the sample fabrication. G.K. performed optical measurement and SEM analysis with support from J.M. H.K. and J.K. performed Python coding for PUF generation and analysis. G.K., Y.M.S., and H.‐H.J. wrote the paper, and all authors contributed to editing.

## Conflicts of Interest

The authors filed one patent application related to the principle and fabrication method.

## Supporting information




**Supporting File 1**: advs77859‐sup‐0001‐SuppMat.docx.

**Supporting File 2**: advs77859‐sup‐0002‐VideoS1.mp4.

**Supporting File 3**: advs77859‐sup‐0003‐VideoS2.mp4.

**Supporting File 4**: advs77859‐sup‐0004‐VideoS3.mp4.

**Supporting File 5**: advs77859‐sup‐0005‐VideoS4.mp4.

## Data Availability

The data that support the findings of this study are available from the corresponding author upon reasonable request.
