# Supplementary material for: Covert Optical Fingerprints Within Overt Structural Colors via Cascaded Cavity Resonances
Source: Adv Sci (Weinh). 2026 Sep 27:e77859. Online ahead of print. doi: 10.1002/advs.77859 (PMC13616307; doi:10.1002/advs.77859)
Supplement: Supplementary file 1 [file ADVS-9999-e77859-s004.docx]

Copyright WILEY-VCH Verlag GmbH & Co. KGaA, 69469 Weinheim, Germany, 2024.

Supporting Information

Covert Optical Fingerprints within Overt Structural Colors via Cascaded Cavity Resonances

Gyurin Kim^1^, Jiyeong Ma^1^, Juhwan Kim^1^, JuHyeong Lee^1^, Heungchan Kwon^1^, Young Min Song^2,3*^, Hyeon-Ho Jeong^1,3,4*^

^1^ Department of Electrical Engineering and Computer Science, Gwangju Institute of Science and Technology, Gwangju, 61005, Republic of Korea

^2^ School of Electrical Engineering, Korea Advanced Institute of Science and Technology, Daejeon, 34141 Republic of Korea

^3^ GIST InnoCORE AI-Nano Convergence Institute for Early Detection of Neurodegenerative Diseases, Gwangju Institute of Science and Technology (GIST), Gwangju 61005, Republic of Korea

^4^ Department of Semiconductor Engineering, Gwangju Institute of Science and Technology, Gwangju, 61005, Republic of Korea

^*^ Corresponding author: [ymsong@kaist.ac.kr](mailto:ymsong@kaist.ac.kr), [jeong323@gist.ac.kr](mailto:jeong323@gist.ac.kr)

**Keywords**: nanophotonics, optical duality, cascaded cavity resonance, short-range correlated disorder, monolithic fabrication

**Supplementary Video S1.** QR-assisted registration and authentication.

**Supplementary Video S2.** Manual finger-rubbing test.

**Supplementary Video S3.** Mechanical abrasion test under repeated pressure loading.

**Supplementary Video S4.** High pressure water exposure test.

**Figure S1.** Optical near-field distribution of cascaded cavities with different HfO_2_ capping layer thickness at reflection resonances.

**Figure S2.** Quantitative comparison of the reflection and scattering responses of the cascaded cavities with varying HfO₂ capping thickness. Simulated (left) and experimental (right) color differences of the reflection and scattering channels relative to the 20 nm HfO₂ capping condition (top). Pearson correlation analysis between the reflection peak wavelength and scattering intensity at 633 nm as a function of HfO₂ capping thickness (bottom), yielding 0.34 and 0.53 for the simulated and experimental responses, respectively.

**Figure S3.** Optical near-field distribution of cascaded cavities with different HfO2 capping layer thickness at scattering resonances.

**Figure S4.** Quantitative analysis of disordered Cu NPs with structural function at real space $g_{2}(R/a)$ and reciprocal space $S\left( qa \right)$ (left panels) based on electron microscopic images at each right panel with different surface areas. $R$ is center-to-center particle distance, $a$ is fixed particle radius, $\rho$ is particle number density, $r_{i}$ is particle centroid position, and $q$ is reciprocal wavevector magnitude.

**Figure S5.** Schematic of the stepwise growth method for various HfO_2_ thicknesses on a single wafer.

**Figure S6.** Fabricated layered cavity (left), nanogap cavity (middle), and cascaded cavity (right). Each structure’s reflection color tunability and near-field stochasticity.

**Figure S7.** Fabrication process for HfO_2_ patterns via photolithography.

**Figure S8.** Stochastic scattering images of 500 different cascaded cavities (top) and generated PUF keys (bottom).

**Fig. S9.** Effect of spatial binning on PUF encoding and spatial autocorrelation of the optical scattering response. Top: Comparison of different image-binning conditions (1 × 1, 2 × 2, 5 × 5, 10 × 10, and 20 × 20 raw pixels), corresponding to nominal object-plane sampling areas ranging from 60 × 60 to 1200 × 1200 nm². Representative electron microscopy regions corresponding to one binned pixel, optical microscope images, red-channel scattering images, generated binary PUF keys, and pairwise Hamming-distance matrices obtained from 500 PUF patterns are shown for each binning condition. The 2 × 2 binning condition (120 × 120 nm²) was selected as the practical encoding condition that preserves local stochastic contrast while limiting excessive spatial averaging. Bottom: Spatial autocorrelation analysis of the raw red-channel scattering images. The normalized two-dimensional autocorrelation functions were calculated for 500 PUF patterns and averaged, followed by radial averaging to obtain the spatial correlation decay. The mean $1/e$ decorrelation distance is 3.67 raw pixels, corresponding to approximately 220 nm in the object plane at a calibrated sampling pitch of 60 nm per pixel.

**Figure S10.** Fabrication process for HfO_2_ patterns on the Cu NPs via shadow mask.

**Figure S11.** A setup for high-pressure water exposure test (left). Measured scattering images, generated PUF keys (middle), and reflection spectra (right) for different water exposure times, ranging from 0 s to 300 s.

**Figure S12.** Acceleration aging test for the cascaded cavities. Representative scattering images (left), original reflection spectra and statistical analyzed data (right) measured after thermal annealing at different temperatures (80–200 °C) and durations (0–4 h).

**Figure S13.** Correspondence between the experimental PUF encoding procedure and FDTD simulation model. The experimental scattering image was acquired using a 50× objective, separated into the red channel, and processed by 2 × 2 pixel binning followed by digitization for PUF key generation. With an object-plane sampling pitch of approximately 60 nm per raw camera pixel, one binned PUF pixel corresponds nominally to a 120 × 120 nm² sampling area. Accordingly, the FDTD model employs a 120 × 120 nm² lateral domain containing non-overlapping spherical Cu nanoparticles with experimentally relevant particle-size variation and a surface coverage of approximately 49.5%. The model represents the local nanoparticle configuration associated with one experimental PUF encoding unit rather than the entire disordered metasurface.

**Supplementary Note 1.** Structural analysis of nanoparticle disorder

The positional correlations of the Cu nanoparticle assemblies were quantitatively analyzed from SEM images to evaluate how rapidly the local nanoparticle arrangement loses structural memory. First, the centroid coordinates of individual Cu nanoparticles were extracted from the SEM images (Fig. S4). The radial pair correlation function, $g_{2}(R)$, was then calculated from these coordinates as[1]

$$g_{2}\left( R \right)=\frac{1}{2\pi R\rho N}\left\langle\sum_{i=1}^{N} \sum_{j\neq i} \delta\left( R-\left| \mathbf{r}_{i}-\mathbf{r}_{j} \right| \right) \right\rangle, (S1)$$

where *N* is the total number of Cu nanoparticles, *ρ* is their number density, *R* is the center-to-center separation between two nanoparticles, $\mathbf{r}_{i}$ and $\mathbf{r}_{j}$ are the position vectors of the *i*-th and *j*-th particles, respectively, and *δ* denotes the Dirac delta function. In the numerical analysis, this expression is evaluated by counting particle pairs within finite radial intervals around *R*. For comparison between nanoparticle assemblies, the distance was normalized by the average particle diameter, *a*, and is therefore presented as *R/a*.

Physically, $g_{2}(R)$ describes how the local packing arrangement around one nanoparticle extends into the surrounding region. At short distances, particle exclusion and local packing produce pronounced deviations of $g_{2}(R)$ from unity. The successive maxima in $g_{2}(R)$ correspond to preferred interparticle separations associated with neighboring coordination shells. Persistence of these oscillatory features over multiple shells therefore indicates positional correlation extending beyond the nearest neighbors. At larger distances, the oscillations progressively diminish and $g_{2}(R)$ approaches unity, indicating that the nanoparticle positions lose positional memory and become effectively uncorrelated. For the cooled-GLAD-grown Cu nanoparticle assemblies, the oscillatory features in $g_{2}(R)$ persist only over the first few coordination shells and rapidly converge toward unity at larger separations. This behavior demonstrates that structural correlation is restricted to the local nanoparticle environment rather than extending throughout the assembly. Consequently, even within a small optical encoding area, the absence of long-range positional constraints allows numerous microscopically distinct nanoparticle configurations to be formed. These local configurational variations provide spatially distinct scattering sites for high-density optical information encoding.

The structure factor was additionally calculated from the same nanoparticle centroid coordinates according to

$$S\left( q \right)=\frac{1}{N}\left| \sum_{j=1}^{N} e^{-i\mathbf{q}\cdot\mathbf{r}_{j}} \right|^{2}, (S2)$$

where **q** is the reciprocal-space wave vector and *q* = $\mid\boldsymbol{q}\mid$. For the isotropic analysis, $S(q)$ was angularly averaged and expressed as a function of the dimensionless reciprocal-space coordinate *qa*. Whereas $g_{2}(R)$ directly probes the persistence of local positional correlations in real space, $S(qa)$ examines whether these correlations develop into long-range periodic ordering in reciprocal space. The absence of sharp Bragg-like peaks in $S(qa)$, together with the rapid disappearance of coordination-shell features in $g_{2}(R)$, demonstrates that the Cu nanoparticle assemblies possess short-range positional correlation without long-range translational order. For comparison with previously reported disorder-engineered optical surfaces, nanoparticle centroid coordinates were extracted from published SEM images with available scale bars, and the same $g_{2}(R)$-based analysis was applied. The persistence of coordination-shell features was compared under the same analysis procedure, providing a consistent basis for the structural comparison summarized in Table S2.

**Supplementary Note 2.** Spatial autocorrelation analysis of stochastic optical scattering

To evaluate the spatial correlation between neighboring optical pixels, spatial autocorrelation analysis was performed directly on the experimentally acquired dark-field scattering images (Fig. S9). A total of 500 cropped RGB scattering images, each consisting of 500 × 500 raw camera pixels, were analyzed. No pixel binning was applied. Because the PUF keys were generated primarily from the red-channel scattering response, the red-channel intensity image, $I(x,y)$, was extracted from each RGB image. The object-plane sampling pitch of one raw camera pixel was approximately 60 nm.

First, the mean intensity was subtracted from each image,

$$I'\left( x,y \right)=I\left( x,y \right)-\overline{I}. (S3)$$

The normalized two-dimensional spatial autocorrelation was then calculated from the mean-subtracted image using a fast Fourier transform,

$$C\left( \Delta x,\Delta y \right)=\mathcal{F}^{-1}\left[ \left| \mathcal{F}\left[ I'\left( x,y \right) \right] \right|^{2} \right], (S4)$$

The autocorrelation was corrected for the number of overlapping pixels at each displacement and normalized such that,

$$C\left( 0,0 \right)=1. (S5)$$

Because the scattering patterns did not exhibit a pronounced directional dependence, the two-dimensional autocorrelation was radially averaged to obtain $C\left( r \right)$. The optical correlation length was defined as the distance at which the normalized correlation decreased to $1/e$,

$$C\left( \xi_{\mathrm{opt}} \right)=\frac{1}{e}. (S6)$$

Across the 500 scattering images, the optical correlation length was$\xi_{\mathrm{opt}}=3.672\pm0.053 \mathrm{raw} \mathrm{pixels}$. Using the object-plane sampling pitch of 60 nm per raw pixel, this corresponds to $\xi_{\mathrm{opt}}=220.3\pm3.2 \mathrm{nm}$. The comparable correlation lengths measured along the horizontal and vertical directions confirmed that the optical correlation was approximately isotropic.

To estimate the effective number of independent bits, the measured correlation length was used as the effective sampling interval in both lateral directions. In this approximation, optical signals separated by approximately one correlation length are treated as effectively independent sampling units, thereby accounting for the spatial correlation between neighboring camera pixels. Thus, the effective number of independent sampling units along one direction was estimated as $136.2$. The effective number of independent bits in a 500 × 500-pixel image was therefore estimated as $N_{\mathrm{eff}}=\left( 500/3.672 \right)^{2}\approx18,541$. The physical area of each analyzed image was 900 μm^2^ and the corresponding effective independent-bit density was therefore $18,541/900 \mu m^{2}\approx2.06\times{10}^{7} \mathrm{bits} mm^{-2}$. These results indicate that the optical correlation extends over approximately 3.7 raw camera pixels, or approximately 220 nm. Treating this correlation length as the effective sampling interval provides an estimated 1.85×10^4^ independent optical degrees of freedom per scattering image. Because the spatial correlation decays continuously rather than defining a discrete boundary between independent pixels, this value represents a correlation-length-based estimate of the effective number of independent optical bits rather than an exact count. This estimate explicitly accounts for the spatial dependence between neighboring camera pixels introduced by the optical imaging system.

**Supplementary Note 3.** Arrhenius extrapolation for lifetime estimation

The thermal stability of the cascaded cavity structures was evaluated using accelerated aging tests performed at elevated temperatures.[2] The temporal evolution of the normalized optical response was fitted using a single-exponential decay function,

$$I\left( t \right)=I_{0}\exp\left( -\frac{t}{\tau} \right), (S7)$$

where $I_{0}$ is the initial optical intensity, $I\left( t \right)$ is the optical intensity after an aging time $t$, and $\tau$ is the characteristic decay time obtained from nonlinear least-squares fitting. In this analysis, *τ* was used as the characteristic lifetime criterion. For a single-exponential decay, $t=\tau$ corresponds to the time at which the optical response decreases to *1/e*, or approximately 36.8%, of its initial value. The corresponding degradation rate constant was defined as

$k=1/\tau. (S8)$

The temperature dependence of the degradation rate was then analyzed using the Arrhenius relationship,

$$k=A\exp\left( -\frac{E_{a}}{k_{B}T} \right), (S9)$$

where $A$ is the pre-exponential factor, $E_{a}$ is the activation energy, $k_{B}$is the Boltzmann constant, and $T$ is the absolute temperature. Taking the natural logarithm gives

$$\ln\left( k \right)=\ln\left( A \right)-\frac{E_{a}}{k_{B}}\frac{1}{T}. (S10)$$

Thus, the activation energy was extracted from the slope of the linear fit of $\ln k$ as a function of $1/T$, yielding $E_{a}$ = 0.568 eV.

The degradation rate at room temperature (20°C, 293.15 K) was subsequently obtained by extrapolating the fitted Arrhenius relationship. The corresponding characteristic lifetime was calculated as

$$\tau_{20^{\circ}C}=\frac{1}{k_{20^{\circ}C}}, (S11)$$

yielding an extrapolated characteristic lifetime of approximately 10.7 years.

The reported value therefore represents the time required for the optical response to decay to *1/e* of its initial value, as extrapolated from accelerated aging measurements. This extrapolation assumes that the same dominant thermally activated degradation mechanism remains operative over the measured and extrapolated temperature ranges.

**Table S1.** Quantitative comparison of the calculated (top) and measured (bottom) CIEDE2000 color differences for the scattering and reflection responses of the cascaded cavities with different HfO₂ capping thicknesses.

| **HfO₂ thickness** | **Scattering**  **(L*, a*, b*)** | **Scattering difference** | **Reflection**  **(L*, a*, b*)** | **Reflection difference** |
| --- | --- | --- | --- | --- |
| 20 nm | (100, 34.62, 40.76) | 0.00 | (100, 49.33, 131.00) | 0.00 |
| 40 nm | (100, 34.53, 40.34) | 0.16 | (100, 76.80, 104.78) | 16.73 |
| 60 nm | (100, 34.28, 40.19) | 0.23 | (100, 100.25, 24.28) | 52.11 |
| 80 nm | (100, 34.10, 40.22) | 0.27 | (100, 1.78, −5.90) | 44.58 |
| 100 nm | (100, 35.41, 41.26) | 0.34 | (100, −16.23, 7.37) | 44.47 |
| 120 nm | (100, 33.92, 41.10) | 0.56 | (100, −15.10, 23.95) | 41.62 |
| 140 nm | (100, 33.99, 41.78) | 0.83 | (100, −9.57, 39.46) | 35.25 |
| 160 nm | (100, 34.17, 42.61) | 1.13 | (100, −3.13, 55.35) | 28.25 |
| 180 nm | (100, 34.45, 43.29) | 1.34 | (100, 4.23, 73.20) | 21.72 |
| 200 nm | (100, 34.81, 43.67) | 1.38 | (100, 13.76, 94.58) | 15.42 |
| 220 nm | (100, 35.27, 43.47) | 1.16 | (100, 26.22, 110.95) | 9.24 |
| 240 nm | (100, 35.68, 42.50) | 0.70 | (100, 40.12, 101.48) | 4.98 |

| **HfO₂ thickness** | **Scattering**  **(L*, a*, b*)** | **Scattering difference** | **Reflection**  **(L*, a*, b*)** | **Reflection difference** |
| --- | --- | --- | --- | --- |
| 20 nm | (100, 74.21, 66.32) | 0.00 | (100, 111.33, −2.66) | 0.00 |
| 40 nm | (100, 78.15, 78.07) | 1.75 | (100, 62.26, −152.59) | 44.50 |
| 60 nm | (100, 55.03, 41.12) | 5.01 | (100, −18.10, −73.74) | 75.49 |
| 80 nm | (100, 65.91, 56.21) | 1.19 | (100, −29.22, −40.06) | 83.27 |
| 100 nm | (100, 51.54, 51.09) | 1.54 | (100, −28.37, −9.59) | 59.23 |
| 120 nm | (100, 40.93, 42.18) | 4.68 | (100, −22.13, 30.58) | 71.24 |
| 140 nm | (100, 18.52, 48.84) | 12.73 | (100, −5.54, 49.59) | 63.66 |
| 160 nm | (100, 18.66, 39.65) | 11.27 | (100, −2.26, 59.46) | 64.47 |
| 180 nm | (100, 22.25, 34.22) | 9.54 | (100, 1.20, 74.42) | 66.53 |
| 200 nm | (100, 30.02, 56.32) | 11.39 | (100, 6.22, 92.50) | 68.25 |
| 220 nm | (100, 33.93, 42.61) | 6.55 | (100, 14.25, 109.39) | 68.19 |
| 240 nm | (100, 33.15, 43.19) | 8.96 | (100, 46.80, 88.54) | 48.92 |

**Table S2.** Comparison of correlation length and surface coverage with previous works.

| **No.** | **fabrication**  **method** | **correlation**  **length (μm)** | **surface**  **coverage (%)** | **Supplementary References** |
| --- | --- | --- | --- | --- |
| 1 | MOCVD | 0.08 | 11.9 | [3] |
| 2 | CVD | 15 | 37.9 | [4] |
| 3 | CVD | 3 | 46.7 | [5] |
| 4 | Spin coating | 120 | 24.4 | [6] |
| 5 | Coating | 2 | 39.3 | [7] |
| 6 | Coating | 0.086 | 24.5 | [8] |
| 7 | Coating | 890 | 43.8 | [9] |
| 8 | PVD | 80 | 45.8 | [10] |
| 9 | PVD | 3 | 14.8 | [11] |
| **this work** | **PVD** | **0.044** | **49.5** |  |

**Table S3.** NIST randomness test for binary sequences generated from the PUF keys.

| **^a^NIST statistical test** | ***^b^p*-value** | **Proportion** | **Result** |
| --- | --- | --- | --- |
| Frequency | 0.4947 | 497/500 | Pass |
| Block frequency | 0.5124 | 495/500 | Pass |
| Cumulative sums | 0.4860 | 497/500 | Pass |
|  | 0.5065 | 495/500 |  |
| Runs | 0.5073 | 495/500 | Pass |
| Longest run | 0.5182 | 497/500 | Pass |
| Serial | 0.5051 | 496/500 | Pass |
|  | 0.5019 | 494/500 |  |
| Approximate entropy | 0.2061 | 492/500 | Pass |

^a^ NIST tests are performed using 500 sequences of 62,500 bits each such that 31,250,000 bits collected from 500 different cascaded layers for the scattering PUFs. The chi-squared (*χ*^2^) distribution is used to compare the goodness-of-fit of the *p*-value distribution of the blocks from the entire bitstream to the expected distribution. The bitstream is considered to random only if the *p*-value ≥0.0001.

^b^ If the proportion exceeds the minimum rate (>480/500) for each test, it is considered a pass.

**Table S4.** Performance comparison with previous optical PUFs.

| **No.** | **material** | **size**  **(mm^2^)** | **bit number** | **degree of freedom** | **information density**  **(bit/mm^2^)** | **degree of integration**  **(PUF/mm^2^)** | **physical decryption time (year)** | **Suppl. ref.** |
| --- | --- | --- | --- | --- | --- | --- | --- | --- |
| 1 | conductor | 0.01225 | 2500 | 864 | 204082 | 81.63265 | 10^1107^ | [8] |
| 2 | conductor | 2.6×10^-4^ | 86 | - | 334000 | 3880 | 10^37.7^ | [12] |
| 3 | conductor | 0.003 | 100 | - | 33333 | 333.3333 | 10^43.9^ | [13] |
| 4 | conductor | 0.0025 | 400 | - | 160000 | 400 | 10^176.8^ | [14] |
| 5 | conductor | 4.3×10^-5^ | 100 | - | 2330000 | 23300 | 10^43.9^ | [15] |
| 6 | conductor | 0.01 | 2500 | - | 250000 | 100 | 10^1107^ | [16] |
| 7 | conductor | 4 | 400 | 319 | 100 | 0.25 | 10^176.8^ | [17] |
| 8 | conductor | 0.075 | 22500 | 875.12 | 300000 | 13.33333 | 10^9966.9^ | [18] |
| 9 | conductor | 0.64 | 1024 | 976.48 | 1600 | 1.5625 | 10^453.2^ | [19] |
| 10 | conductor | 1 | 1024 | 612.5525 | 1024 | 1 | 10^453.2^ | [20] |
| 11 | conductor | 2×10^-5^ | 384 | 348 | 19200000 | 50000 | 10^170^ | [21] |
| 12 | conductor | - | 262144 | - | - | - | 10^116127.8^ | [22] |
| 13 | conductor | 400 | 896 | 774 | 2.24 | 0.0025 | 10^396.5^ | [23] |
| 14 | conductor | 2.028 | 2048 | 17.2559 | 1010 | 0.4931 | 10^906.8^ | [10] |
| 15 | dielectric | 1.011 | 45 | 84.16783 | 45 | 0.98912 | 10^19.5^ | [24] |
| 16 | dielectric | 23.086 | 15200 | 190 | 658 | 0.04332 | 10^6733.1^ | [25] |
| 17 | dielectric | 169 | 64 | 345 | 0.38 | 0.00592 | 10^27.9^ | [26] |
| 18 | dielectric | - | - | - | - | - | - | [27] |
| 19 | dielectric | 0.003 | 144 | 20.4 | 48000 | 333.3333 | 10^63.3^ | [28] |
| 20 | dielectric | 0.01 | 36 | 297 | 3600 | 100 | 10^15.5^ | [29] |
| 21 | dielectric | 0.4 | 256 | 216 | 640 | 2.5 | 10^113^ | [30] |
| 22 | dielectric | 4.986 | 256 | 103 | 51 | 0.20056 | 10^113^ | [6] |
| 23 | dielectric | 100 | 2500 | - | 25 | 0.01 | 10^1107^ | [31] |
| 24 | dielectric |  | 100 | 18.2 | - | - | - | [32] |
| 25 | dielectric | 0.25 | 400 | 370 | 1600 | 4 | 10^176.8^ | [33] |
| 26 | dielectric | 1.248 | 25 | - | 20 | 0.80128 | 10^10.6^ | [34] |
| 27 | dielectric | 1×10^-4^ | - | 4220 | 42200000 | 10000 | - | [3] |
| 28 | dielectric | 3.24 | 10000 | 188.65 | 3086 | 0.30864 | 10^4429.5^ | [35] |
| 29 | dielectric | 0.03063 | 62500 | 1371.73 | 2040816 | 32.65306 | 10^27686.7^ | [36] |
| 30 | dielectric | - | - | - | - | - | - | [37] |
| 31 | dielectric | 12.25 | 928 | 1750 | 76 | 0.08163 | 10^410.7^ | [38] |
| 32 | dielectric | 0.0023 | 1024 | 13350.18 | 444444 | 434.0278 | 10^453.2^ | [9] |
| 33 | dielectric | 1 | 1024 | 541 | 1024 | 1 | 10^453.2^ | [39] |
| 34 | dielectric | 9×10^-4^ | 2700 | - | 3000000 | 1110 | 10^1200^ | [40] |
| 35 | semiconductor | 55.369 | 256 | 120 | 4.6 | 0.01806 | 10^113^ | [41] |
| 36 | semiconductor | 4×10^-6^ | 64 | 62.5 | 16000000 | 250000 | 10^27.9^ | [4] |
| 37 | semiconductor | 1.123 | 192 | 69 | 171 | 0.89047 | ^-^ | [42] |
| 38 | semiconductor | 0.01 | 100 | - | 10000 | 100 | ^-^ | [5] |
| 39 | semiconductor | 1 | 105625 | 566 | 105625 | 1 | 10^46790.8^ | [7] |
| 40 | semiconductor | 2.3×10^-5^ | 10000 | 626 | 442000000 | 44200 | 10^4430^ | [43] |
| 41 | semiconductor | 100 | 400 | 305.63 | 4 | 0.01 | 10^176.8^ | [44] |
| 42 | semiconductor | 4 | 49 | 97656.18 | 12.25 | 0.25 | 10^21.2^ | [45] |
| 43 | semiconductor | - | 10000 | - | - | - | 10^4429.5^ | [46] |
| 44 | semiconductor | 2.5×10^-7^ | 4 | - | 16000000 | 4000000 | 10^1.3^ | [47] |
| 45 | semiconductor | 250 | 1600 | - | 6.4 | 0.004 | 10^708.3^ | [48] |
| 46 | semiconductor | 0.0016 | 100 | 157 | 62500 | 625 | 10^43.9^ | [49] |
| 47 | semiconductor | 0.0025 | 400 | 825.73 | 160000 | 400 | 10^176.8^ | [50] |
| 48 | semiconductor | 0.0016 | 400 | - | 250000 | 625 | 10^176.8^ | [51] |
| **this work** | **conductor** | **0.00117** | **62500** | **3905.25** | **69400000** | **854.9639** | 10**^27686.7^** |  |

**Supplementary References**

[1]	K. Vynck, R. Pierrat, R. Carminati, et al., “Light in correlated disordered media,” *Reviews of Modern Physics* 95, no. 4 (2023): 45003. https://doi.org/10.1103/RevModPhys.95.045003

[2]	J. Allison, P. Anderson, E. Aranas, et al., “Laser writing in glass for dense, fast and efficient archival data storage,” *Nature* 650, no. 8102 (2026): 606–612. https://doi.org/10.1038/s41586-025-10042-w

[3]	Q. Li, F. Chen, J. Su, et al., “Quantum Physical Unclonable Function Based on Multidimensional Fingerprint Features of Single Photon Emitters in Random AlN Nanocrystals,” *Advanced Functional Materials* 35, no. 9 (2025): 2416216. https://doi.org/10.1002/adfm.202416216

[4]	A. Alharbi, D. Armstrong, S. Alharbi, and D. Shahrjerdi, “Physically Unclonable Cryptographic Primitives by Chemical Vapor Deposition of Layered MoS2,” *ACS Nano* 11, no. 12 (2017): 12772–12779. https://doi.org/10.1021/acsnano.7b07568

[5]	P. Chen, D. Li, Z. Li, et al., “Programmable Physical Unclonable Functions Using Randomly Anisotropic Two-Dimensional Flakes,” *ACS Nano* 17, no. 23 (2023): 23989–23997. https://doi.org/10.1021/acsnano.3c08740

[6]	A. Esidir, N. Kayaci, N. B. Kiremitler, et al., “Food-Grade Physically Unclonable Functions,” *ACS Applied Materials & Interfaces* 15, no. 35 (2023): 41373–41384. https://doi.org/10.1021/acsami.3c09035

[7]	J. Zhang, Y. Liu, C. Njel, S. Ronneberger, N. V Tarakina, and F. F. Loeffler, “An all-in-one nanoprinting approach for the synthesis of a nanofilm library for unclonable anti-counterfeiting applications,” *Nature Nanotechnology* 18, no. 9 (2023): 1027–1035. https://doi.org/10.1038/s41565-023-01405-3

[8]	G. Kim, D. Kim, J. Lee, et al., “Quasi-ordered plasmonic metasurfaces with unclonable stochastic scattering for secure authentication,” *Nature Communications* 16, no. 1 (2025): 6269. https://doi.org/10.1038/s41467-025-61570-y

[9]	U. Jung, C.-J. Beak, K. Kim, J.-H. Na, and S.-H. Lee, “Scalable Photo-Responsive Physical Unclonable Functions via Particle Kinetics,” *ACS Nano* 18, no. 40 (2024): 27642–27653. https://doi.org/10.1021/acsnano.4c09080

[10]	S. Gandla, J. Yoon, C. Yang, H. Lee, W. Park, and S. Kim, “Random laser ablated tags for anticounterfeiting purposes and towards physically unclonable functions,” *Nature Communications* 15, no. 1 (2024): 7592. https://doi.org/10.1038/s41467-024-51756-1

[11]	F. Foadi, I. Torun, M. Sakir, M. Kalay, J. Ye, and M. S. Onses, “Multi-Level Hybrid Anti-Counterfeit Labels via Solid-State Dewetting Under Spatial Confinement,” *Advanced Materials Technologies* 10, no. 23 (2025): e01195. https://doi.org/10.1002/admt.202501195

[12]	Y. Lu, H. Chen, H. Cheng, H. Qiu, C. Jiang, and Y. Zheng, “Plasmonic Physical Unclonable Function Labels Based on Tricolored Silver Nanoparticles: Implications for Anticounterfeiting Applications,” *ACS Applied Nano Materials* 5, no. 7 (2022): 9298–9305. https://doi.org/10.1021/acsanm.2c01622

[13]	Y. Lu, H. Cheng, G.-C. Li, et al., “Dynamic Cryptography through Plasmon-Enhanced Fluorescence Blinking,” *Advanced Functional Materials* 32, no. 30 (2022): 2201372. https://doi.org/10.1002/adfm.202201372

[14]	Z. Tang, X. Liu, X. Liu, et al., “Unclonable Anti-Counterfeiting Labels Based on Plasmonic-Patterned Nanostructures,” *Advanced Engineering Materials* 24, no. 9 (2022): 2101701. https://doi.org/10.1002/adem.202101701

[15]	J. D. Smith, M. A. Reza, N. L. Smith, et al., “Plasmonic Anticounterfeit Tags with High Encoding Capacity Rapidly Authenticated with Deep Machine Learning,” *ACS Nano* 15, no. 2 (2021): 2901–2910. https://doi.org/10.1021/acsnano.0c08974

[16]	Y. Gu, C. He, Y. Zhang, L. Lin, B. D. Thackray, and J. Ye, “Gap-enhanced Raman tags for physically unclonable anticounterfeiting labels,” *Nature Communications* 11, no. 1 (2020): 516. https://doi.org/10.1038/s41467-019-14070-9

[17]	Z. Wang, H. Wang, F. Li, X. Gao, and Y. Shao, “Physical Unclonable Functions Based on Photothermal Effect of Gold Nanoparticles,” *ACS Applied Materials & Interfaces* 16, no. 14 (2024): 17954–17964. https://doi.org/10.1021/acsami.3c18270

[18]	J. Duan, G. Li, Y. Xiong, et al., “Scalable plasmonic physical unclonable functions empowered by a multi-dimensional expanding strategy,” *Advanced Photonics Nexus* 4, no. 1 (2024): 16003. https://doi.org/10.1117/1.APN.4.1.016003

[19]	Y. A. Kim, C. Moon, S. Kim, and B. Lim, “Web-Like Silver Nanowire Networks via Marangoni-Induced Self-Assembly for Physically Unclonable Tags,” *Advanced Functional Materials* 35, no. 49 (2025): 8556. https://doi.org/10.1002/adfm.202508556

[20]	Y. Kim, K. Min, J. H. Lim, et al., “Laser-Induced Graphene-Based Dual-Functional Optical PUFs for Hierarchical Authentication,” *Advanced Materials Technologies* n/a, no. n/a (2026): e02370. https://doi.org/10.1002/admt.202502370

[21]	J.-K. Kwak, C. Moon, H.-B. Yu, S. Kim, and D.-H. Kim, “All-Optical Reconfigurable Physical Unclonable Function for Sustainable Security,” *Advanced Materials* n/a, no. n/a (2026): e21712. https://doi.org/10.1002/adma.202521712

[22]	Y. Gao, K. Ge, Z. Zhang, et al., “Fine Optimization of Colloidal Photonic Crystal Structural Color for Physically Unclonable Multiplex Encryption and Anti-Counterfeiting,” *Advanced Science* 11, no. 20 (2024): 2305876. https://doi.org/10.1002/advs.202305876

[23]	H. Im, J. Yoon, B. So, et al., “Four-Dimensional Physical Unclonable Functions and Cryptographic Applications Based on Time-Varying Chaotic Phosphorescent Patterns,” *ACS Nano* 18, no. 18 (2024): 11703–11716. https://doi.org/10.1021/acsnano.3c12432

[24]	J. H. Kim, S. Jeon, J. H. In, et al., “Nanoscale physical unclonable function labels based on block copolymer self-assembly,” *Nature Electronics* 5, no. 7 (2022): 433–442. https://doi.org/10.1038/s41928-022-00788-w

[25]	M. S. Kim, and G. J. Lee, “Visually Hidden, Self-Assembled Porous Polymers for Optical Physically Unclonable Functions,” *ACS Applied Materials & Interfaces* 15, no. 3 (2023): 4477–4486. https://doi.org/10.1021/acsami.2c18737

[26]	M. S. Kim, G. J. Lee, J. W. Leem, S. Choi, Y. L. Kim, and Y. M. Song, “Revisiting silk: a lens-free optical physical unclonable function,” *Nature Communications* 13, no. 1 (2022): 247. https://doi.org/10.1038/s41467-021-27278-5

[27]	G. Park, H. Park, J. M. Wolska, J. G. Park, and D. K. Yoon, “Racemized photonic crystals for physical unclonable function,” *Materials Horizons* 9, no. 10 (2022): 2542–2550. https://doi.org/10.1039/D2MH00710J

[28]	K. Kim, S.-U. Kim, M.-Y. Choi, M. H. Saeed, Y. Kim, and J.-H. Na, “Voxelated opto-physically unclonable functions via irreplicable wrinkles,” *Light: Science & Applications* 12, no. 1 (2023): 245. https://doi.org/10.1038/s41377-023-01285-1

[29]	S. M. Park, G. Park, and D. K. Yoon, “Paintable Physical Unclonable Functions using DNA,” *Advanced Materials* 35, no. 35 (2023): 2302135. https://doi.org/10.1002/adma.202302135

[30]	G. Park, Y.-S. Choi, S. J. Kwon, and D. K. Yoon, “Planar Spin Glass with Topologically Protected Mazes in the Liquid Crystal Targeting for Reconfigurable Micro Security Media,” *Advanced Materials* 35, no. 36 (2023): 2303077. https://doi.org/10.1002/adma.202303077

[31]	J. Wu, X. Liu, X. Liu, et al., “A High-Security mutual authentication system based on structural color-based physical unclonable functions labels,” *Chemical Engineering Journal* 439 (2022): 135601. https://doi.org/10.1016/j.cej.2022.135601

[32]	J. Yang, M. Feng, J. Wang, et al., “Bionic Micro-Texture Duplication and RE3+ Space-Selective Doping of Unclonable Silica Nanocomposites for Multilevel Encryption and Intelligent Authentication,” *Advanced Materials* 35, no. 49 (2023): 2306003. https://doi.org/10.1002/adma.202306003

[33]	Z. Wang, H. Wang, P. Wang, and Y. Shao, “Robust Optical Physical Unclonable Function Based on Total Internal Reflection for Portable Authentication,” *ACS Applied Materials & Interfaces* 16, no. 21 (2024): 27926–27935. https://doi.org/10.1021/acsami.4c03283

[34]	A. Esidir, M. Ren, S. Pekdemir, et al., “Structurally Colored Physically Unclonable Functions with Ultra-Rich and Stable Encoding Capacity,” *Advanced Functional Materials* 35, no. 12 (2025): 2417673. https://doi.org/10.1002/adfm.202417673

[35]	X. Lin, D. Song, C. Xu, et al., “Bio-Inspired Design of Quasi-Ordered Structural Color via Stress-Driven Reconfiguration Enables Ultra-Secure and Scalable Unclonable Application,” *Advanced Materials* 38, no. 17 (2026): e20440. https://doi.org/10.1002/adma.202520440

[36]	Y. Li, S. Deng, Y. Li, et al., “Multilevel Intelligent Anti-Counterfeiting Label with Spatially Selective Dynamic Aurora Response and 3D Mesoscopic Physical Unclonable Function Fingerprint,” *Small* 21, no. 24 (2025): 2502819. https://doi.org/10.1002/smll.202502819

[37]	Z. He, B. Cao, Y. Zhang, et al., “Reversible and Reconfigurable Optical Physical Unclonable Functions with Elastic Liquid Crystal Scatterers,” *Nano Letters* 25, no. 43 (2025): 15542–15549. https://doi.org/10.1021/acs.nanolett.5c03711

[38]	S. Nocentini, U. Rührmair, M. Barni, D. S. Wiersma, and F. Riboli, “All-optical multilevel physical unclonable functions,” *Nature Materials* 23, no. 3 (2024): 369–376. https://doi.org/10.1038/s41563-023-01734-7

[39]	Y. Kim, J. Lim, J. H. Lim, et al., “Reconfigurable Multilevel Optical PUF by Spatiotemporally Programmed Crystallization of Supersaturated Solution,” *Advanced Materials* 35, no. 22 (2023): 2212294. https://doi.org/10.1002/adma.202212294

[40]	M. R. Carro-Temboury, R. Arppe, T. Vosch, and T. J. Sørensen, “An optical authentication system based on imaging of excitation-selected lanthanide luminescence,” *Science Advances* 4, no. 1 (2026): e1701384. https://doi.org/10.1126/sciadv.1701384

[41]	J. W. Leem, M. S. Kim, S. H. Choi, et al., “Edible unclonable functions,” *Nature Communications* 11, no. 1 (2020): 328. https://doi.org/10.1038/s41467-019-14066-5

[42]	N. B. Kiremitler, A. Esidir, G. A. Drake, et al., “Tattoo-Like Multi-Color Physically Unclonable Functions,” *Advanced Optical Materials* 12, no. 12 (2024): 2302464. https://doi.org/10.1002/adom.202302464

[43]	H. Guo, Y. Qin, Z. Wang, et al., “Multilevel Encoding Physically Unclonable Functions Based on The Multispecies Structure in Diamonds,” *Advanced Functional Materials* 34, no. 15 (2024): 2304648. https://doi.org/10.1002/adfm.202304648

[44]	Y. Jing, F. Jiao, Z. Wang, et al., “Wavelength-Multiplexed PUFs Through Single-Host Multicolor Switching in Cs2NaTbCl6: Eu3+ Double Perovskites,” *Advanced Materials* 38, no. 16 (2026): e15271. https://doi.org/10.1002/adma.202515271

[45]	B. Lyu, Y. Ouyang, D. Gao, X. Wan, and X. Bao, “Multilevel and Flexible Physical Unclonable Functions for High-End Leather Products or Packaging,” *Small* 21, no. 3 (2025): 2408574. https://doi.org/10.1002/smll.202408574

[46]	J. Huang, X. Jin, X. Yang, T. Zhao, H. Xie, and P. Duan, “Near-Infrared Circularly Polarized Luminescent Physical Unclonable Functions,” *ACS Nano* 18, no. 24 (2024): 15888–15897. https://doi.org/10.1021/acsnano.4c03136

[47]	Z. Man, C. Dong, J. Bian, Z. Lu, Y. Lu, and W. Zhang, “Optically Readable, Physically Unclonable Subwavelength Pixel via Multicolor Quantum Dot Printing for Anticounterfeiting,” *Nano Letters* 24, no. 23 (2024): 7019–7024. https://doi.org/10.1021/acs.nanolett.4c01463

[48]	Y.-W. Hu, Q. Cao, S.-Y. Song, et al., “Time-dependent physical unclonable functions by long-lived triplet excitons in carbon dots,” *Light: Science & Applications* 14, no. 1 (2025): 283. https://doi.org/10.1038/s41377-025-01940-9

[49]	M. Sandomirskii, E. Petrova, P. Kustov, et al., “Spectral physical unclonable functions: downscaling randomness with multi-resonant hybrid particles,” *Nature Communications* 16, no. 1 (2025): 5097. https://doi.org/10.1038/s41467-025-60121-9

[50]	J. Ahn, T. Park, T. Kang, et al., “Nanoseed-based physically unclonable function for on-demand encryption,” *Science Advances* 11, no. 17 (2026): eadt7527. https://doi.org/10.1126/sciadv.adt7527

[51]	K. Wang, J. Shi, W. Lai, et al., “All-silicon multidimensionally-encoded optical physical unclonable functions for integrated circuit anti-counterfeiting,” *Nature Communications* 15, no. 1 (2024): 3203. https://doi.org/10.1038/s41467-024-47479-y
